# Complex Permittivity and Electromagnetic Interference Shielding Effectiveness of OPEFB Fiber-Polylactic Acid Filled with Reduced Graphene Oxide

**DOI:** 10.3390/ma13204602

**Published:** 2020-10-16

**Authors:** Ismail Ibrahim Lakin, Zulkifly Abbas, Rabaah Syahidah Azis, Ibrahim Abubakar Alhaji

**Affiliations:** 1 Department of Physics, Faculty of Science, University Putra Malaysia (UPM), Serdang 43400, Malaysia; gs51771@student.upm.edu.my (I.I.L.); rabaah@upm.edu.my (R.S.A.); gs54099@student.upm.edu.my (I.A.A.); 2 Material Synthesis and Characterization Laboratory, Institute of Advanced Technology (ITMA), University Putra Malaysia (UPM), Serdang 43400, Malaysia

**Keywords:** fiber, polymer, dielectric properties, reduced graphene oxide, EMI shielding effectiveness

## Abstract

This study was aimed at fabricating composites of polylactic acid (PLA) matrix-reinforced oil palm empty fruit bunch (OPEFB) fiber filled with chemically reduced graphene oxide (rGO). A total of 2–8 wt.% rGO/OPEFB/PLA composites were characterized for their complex permittivity using an open-ended coaxial probe (OEC) technique. The shielding efficiency properties were calculated using the measured transmission (S_21_) and the reflection (S_11_) coefficient results. All the measurements and calculations were performed in the 8–12 GHz frequency range. The morphological and microstructural study included X-ray diffraction (XRD), field emission scanning electron microscopy (FE-SEM), and Fourier transform infrared spectroscopy (FTIR). The results indicated that the incorporation of rGO as filler into the composites enhanced their complex permittivity properties. The composites showed a total shielding efficiency (SE_T_) of about 31.2 dB at a frequency range of 8–12 GHz, which suggests their usefulness for microwave absorption.

## 1. Introduction

In this contemporary world of information technology (IT), we are surrounded by telecommunications and electronic devices, and their use is unavoidable. Electromagnetic interference (EMI) is an unwanted by-product of rapid electronic systems and telecommunication devices. This radiation can result in improper operation of other equipment [1,2,3]. Efforts have been made to minimize emissions of an electromagnetic nature by using EMI absorbing materials [4]. These materials attenuate signals by absorbing and/or reflecting the radiation energy [5]. Several materials, such as metals, carbon materials (carbon nanotubes, and activated carbons, etc.), and their composites, are excellent candidates for EMI absorbing applications [6,7]. For this purpose, conventional metal shielding materials are used for a long time and serve as the most effective EMI shield. However, they have several drawbacks, such as being corrosive, heavyweight, complicated processing, and additionally shielding is by reflection, which will minimally contribute to the reduction of EM pollution [8].

Conductive polymer composites (CPCs) have attracted both research and industrial interest for many decades due to their high flexibility, wideband electrical conductivity, secure processing, and low cost, which make them promising candidates for EMI shielding applications. They are considered a significant group of relatively cost-effective materials to satisfy a wide range of engineering applications such as electrically conductive adhesives, antistatic coatings and films, sensors, electromagnetic and electrical energy storage devices, and an efficient EMI shielding material [9,10]. Excellent shielding effectiveness has been reported on CPCs filled with carbon materials such as carbon fibers [11], carbon black [12], carbon nanotubes (CNTs) [13], and graphite [14] in terms of their intrinsic properties such as aspect ratios, electrical conductivities, and dielectric properties. Most commercial polymers, like polyethylene, polypropylene, and polystyrene, are not biodegradable. Polylactic acid (PLA), as thermoplastic polyester, is flexible and is produced mostly from annually renewable materials. PLA has become of high-interest due to its adaptability and suitability in many production techniques such as injection molding, extrusion, and thermoforming.

Moreover, PLA has high strength, elasticity modulus, stiffness, and is a fully biodegradable matrix. The emergence of natural fiber composites has fascinated research attention in recent years because, in terms of weight reduction, fibers can replace conventional reinforcing materials [15]. PLA reinforced with pineapple leaf [16], kenaf [17], oil palm empty fruit bunch fiber (OPEFB) [18], and other natural fibers have been studied.

Recently, different materials with graphene particles were investigated and showed exciting results in electromagnetic absorption. Due to their excellent properties, graphene materials have been used in multifunctional polymer composites as a filler [19,20]. Graphene-polymethyl methacrylate microcellular nanocomposite foams showed an EMI shielding effectiveness up to 19 dB at a frequency range of 8–12 GHz with the help of multiple reflections and scattering incident microwaves into the foam samples [21]. Epoxy resin polymer filled with reduced graphene oxide attained a value of 25. 748 dB at 12 GHz for the 5 wt.% reduced graphene [22]. PLA/GNP nanocomposites exhibit total Shielding effectiveness SE_T_ of 14.6 and 15.5 dB for PL15 in bands C- and X, respectively [23].

In this study, chemically reduced graphene oxide (rGO) was synthesized. A total of 2–8 wt.% rGO content was incorporated into the OPEFB/PLA matrix to study the dielectric properties of the composites. EMI shielding performance of rGO/OPEFB/PLA composites was determined in terms of reflection, absorption shielding effectiveness. All the measurements were performed at the 8–12 GHz frequency range. The dielectric measurement of the samples was carried out using the Open-ended coaxial (OEC) probe technique. XRD and FESEM were used to study the physical, chemical, and surface morphology of the composites. The 8% rGO composite gave an optimum $(SE_{T}$) value of 31.2 dB, which is suitable for EMI shielding.

## 2. Material and Methods

### 2.1. Materials

OPEFB fiber was obtained from, Dengkil, Selangor, Malaysia. PLA was bought under the trade name polylactide resin 3052D from nature works LLC (Minnetonka, MN, USA). Graphite powder and reduced graphene oxide (rGO) (synthesized using ammonia (NH_3_) as a reducing agent obtained from Sigma Aldrich (Sarasota, FL, USA)).

### 2.2. Synthesis of rGO

**Figure 1:**
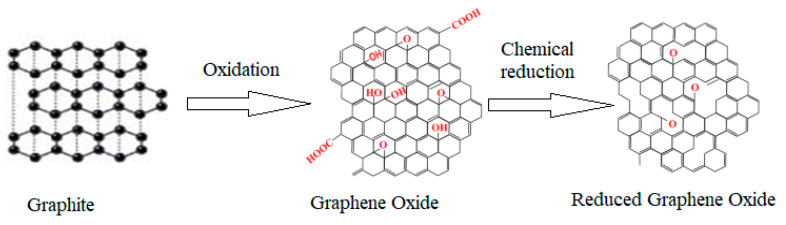
rGO fabrication route.

The Staudenmaier method was used to synthesize Graphite Oxide (GO) [20]. The reduction of GO to rGO was carried out by putting approximately 400 mg of GO into a thimble for cellulose extraction 30 mm × 100 mm and then put in the extraction unit Soxhlet. 150 mL of 30% (NH_3_) solution, which served as a reducing agent, was used. The sample was heated set at 80 °C for an exposure time of 6 h. The synthesis process is presented in Figure 1.

### 2.3. Fabrication of rGO/OPEFB/PLA Composites

**Figure 2:**
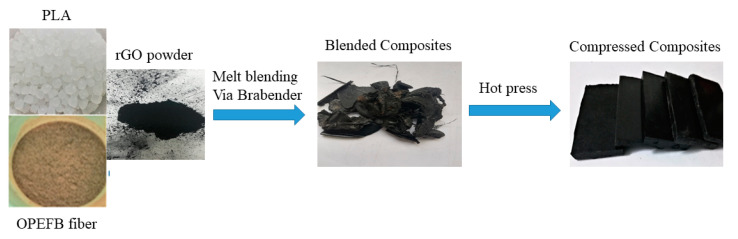
Fabrication of rGO/OPEFB/PLA composites.

The OPEFB fibers were soaked in distilled water for 24 h to remove the wax layer of fibers. The fibers were rinsed with acetone and dried in an oven at 80 °C for 6 h to reduce the moisture. The dried fibers were crushed into powder using a crusher machine (Model JL1000-55, JLNE, Jiangsu, China), which was then sifted 100 µm by a laboratory sieve (Endecotts, London, UK). The rGO/OPEFB/PLA composites were manufactured by mixing 2%, 4%, 6%, and 8% mass percentages of rGO with OPEFB fiber and PLA at a fixed mass ratio of 3:7. The PLA was melted in Brabenda Internal Mixer (Model 815651, GmbH & CO. KG, Duisburg, Germany) for 2 min then the OPEFB followed by the rGO powders were added and continued blending for another 12 min at 160 °C with 50 rpm of rotor speed. The composites samples were compressed separately to a thickness of 6 mm, with a hydraulic press (Fred S. Carver part No.:973110A, Wabash, IN, USA) in sample holders at 4 tons, to eliminate air gaps inside the sample likely to influence the results. Figure 2 shows the fabrication of rGO/OPEFB/PLA composites process.

### 2.4. Characterization

#### 2.4.1. Structure and Composition

Field Emission Scanning Electron microscopy (FE-SEM) was used to examine the morphology of rGO/OPEFB/PLA composites. For morphological characterization, the Nova NanoSEM (FEI, Eindhoven, The Netherlands) operated at an accelerating voltage of 20 kV was used. Samples were put over a carbon tape on the stub for scanning. The gold coating was applied to the sample. X-ray diffraction (XRD) measurement was conducted through a Shimadzu XRD 600 diffractometer (Tokyo, Japan) with a nickel-filtered Cu-Kα (α = 0.1542 nm) beam performed at 30 kV voltage and 30 mA current. The composites were scanned within the range of scattering angles of 2Ɵ of 10° to 80° at the rate of 2°/min. Fourier transforms infrared (FTIR) analysis was carried out through the Perkin Elmer Model 100 series (Waltham, MA, USA). The samples were registered in the wavenumber range from 400 to 4000 cm^−1^.

#### 2.4.2. Dielectric Properties

**Figure 3:**
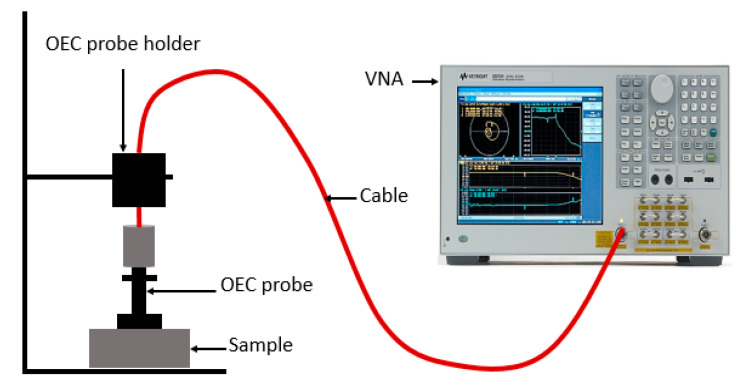
Complex permittivity measurement using OEC.

The measurements for the complex permittivity of the composites were carried out using the OEC probe HP85071C technique (Agilent, Santa Clara, CA, USA). The probe was connected to an HP8720B Vector Network Analyzer (VNA) via a high-precision coaxial test cable [24]. The OEC probe is particularly suited for the measurements of complex permittivity of liquid. It can be used for solid but special attention should be paid concerning samples flatness and the contact between the sample and the probe. A standard one-port, short-air-water calibration was performed, and a reference standard material (polytetrafluoroethylene) was characterized to validate the accuracy of the calibration. As illustrated in Figure 3, the OEC probe was then firmly positioned on the flat surface of the powdered samples to determine complex permittivity using the software installed on the VNA. All the measurements were performed in the 8–12 GHz frequency range.

#### 2.4.3. Shielding Effectiveness

The combined result of attenuation due to absorption, reflection, and multiple internal reflection losses at the interfaces of a material, encountered by incident EM wave is known as total shielding effectiveness (*SE_T_*) and can be given as:

(1) $$SE_{T}=\left(SE_{R}+SE_{A}+SE_{M}\right)$$

A two-port VNA reflected and transmitted wave, also known as S-parameters ($S_{11}$ and $S_{21}$), is related to reflectance (${\left|S_{11}\right|}^{2})$, transmittance (${\left|S_{21}\right|}^{2}$), and absorbance (A=1−R−T). The shielding performance is calculated by the logarithmic proportion of incident and transmitted EM radiation, and can be expressed [25] as:

(2) $$SE_{A}=10\log_{1o}\left(\frac{1-R}{T}\right)=10\log_{10}\left(\frac{1-{\left|S_{11}\right|}^{2}}{{\left|S_{21}\right|}^{2}}\right)$$

(3) $$SE_{R}=10\log_{10}\left(\frac{1}{1-R}\right)=10\log_{10}\left(\frac{1}{1-{\left|S_{11}\right|}^{2}}\right)$$

(4) $$SE_{T}=10\log_{10}\left(\frac{1}{T}\right)=10\log_{10}\left(\frac{1}{{\left|S_{21}\right|}^{2}}\right)$$

## 3. Results and Discussion

### 3.1. Field Emission Scanning Electron Microscopy

**Figure 4:**
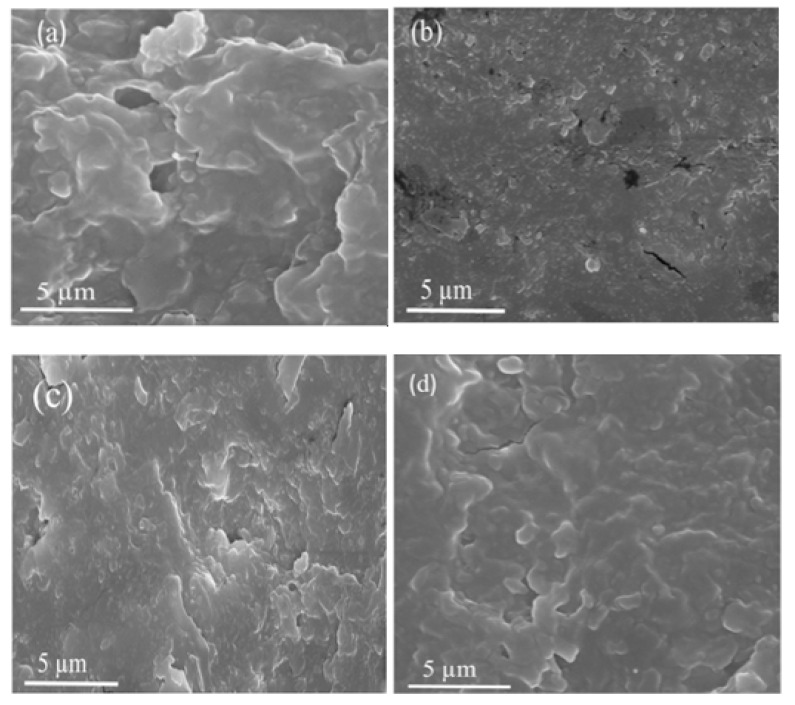
FE-SEM micrographs of (**a**) rGO, (**b**) 2 wt.%, (**c**) 4 wt.%, and (**d**) 8 wt.% rGO fillers.

FE-SEM images were analyzed for blend morphology and additive distribution. The FE-SEM results for the rGO and the rGO/OPEFB/PLA substrates at various rGO contents (2%, 4%, and 8% rGO) are presented in Figure 4. It is observed that (Figure 4a) the rGO is irregular, and has many folded layers at the edges [26]. The flake-like layers are continuously cross-linked in a brittle textured form without any other crystallized particle phase [27]. The 2 wt.% of rGO was homogeneously dispersed in the PLA reinforced OPEFB fiber composite (Figure 4b). The rGO dispersion (Figure 4c,d) indicates a significant difference between the 4% and 8% percentage rGO. It is observed that (Figure 4d) the rGO filler was distributed in the OPEFB/PLA matrix in which the irregular, wrinkled, and folded layered at the edges features of rGO powder resurfaces. The morphological figures indicate that rGO filler did react with the OPEFB/PLA substrates.

### 3.2. X-ray Diffraction

**Figure 5:**
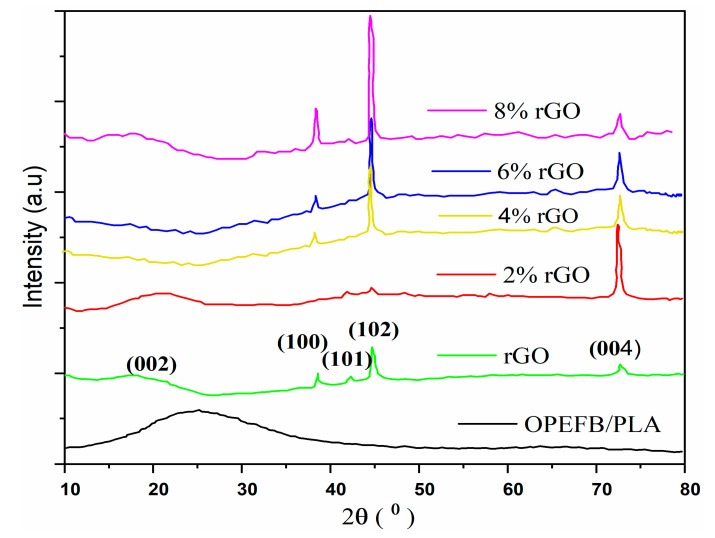
XRD patterns of OPEFB/PLA, rGO, and rGO/OPEFB/PLA at various percentages of rGO filler.

XRD diffractograms of rGO, OPEFB/PLA, and rGO/OPEFB/PLA composites are presented in Figure 5. The XRD patterns of pure OPEFB/PLA showed a broad diffraction peak at approximately 2θ ≈ 17.42° to 30.21°. The OPEFB/PLA showed no characteristic peak, which indicates that PLA [28] and OPEFB [29] has an amorphous structure. The broad peaks of OPEFB/PLA suggest carbon-based and cellulose type material [18]. The XRD spectra of the reduced rGO had several diffraction peaks at 2θ ≈ 18.85°, 39.06°, 42.67°, 45.74°, and 73.27° parts corresponding to the (002), (100), (101), (102), and (004) hexagonal carbon structure, respectively (JCPDS no. 19-0629) [30]. The highest peak of rGO appeared at 45.74°, indicating a high degree of crystallinity. It is observed that with the addition of 2–8 wt.% rGO, the intensity of the OPEFB/PLA matrix increased significantly, which in turn increased the composite crystallinity. This increase can be attributed to rGO nucleating effect in PLA [23]. The rGO/OPEFB/PLA composites with 2%, 4%, 6%, and 8% rGO contents also exhibit the highest diffraction peaks at 2θ ≈ 73.16°, 45.39°, 45.70° and 45.94°, respectively.

### 3.3. Fourier Transform Infrared

**Figure 6:**
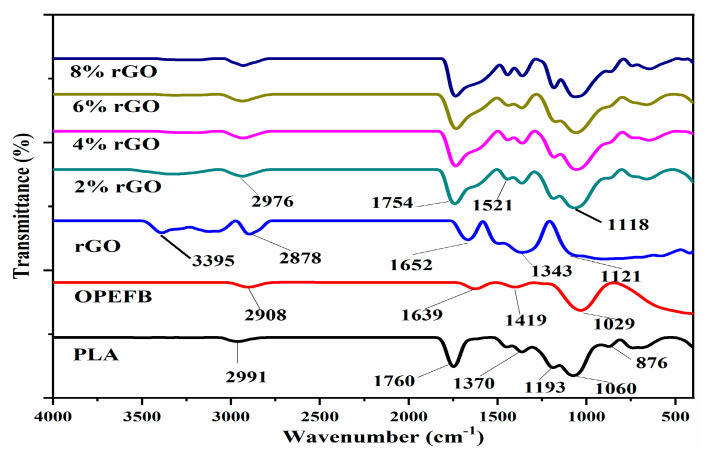
FTIR of OPEFB fiber, PLA, rGO, and rGO/OPEFB/PLA at various percentages of rGO loadings.

The FTIR spectra for OPEFB fiber, PLA, rGO, and OPEFB/PLA matrix filled with various (2%, 4%, 6%, and 8%) percentages of rGO is shown in Figure 6. The characteristic band at 2991 cm^−1^ to 2976 cm^−1^ corresponds to C–H stretching vibrations, 1652 cm^−1^ (C=C skeletal vibration), 1521 cm^−1^ (C–C stretching), 1121 cm^−1^ (C–O–C stretching), and 874 cm^−1^ (C=O stretching) [31]. Furthermore, the absorption bands at 1343 cm^−1^ to 1419 cm^−1^ correspond to C–OH stretching, 2927 cm^−1^ C–H stretching, and 3395 cm^−1^ (O–H vibration) [32]. A shift in characteristic bands (1754 cm^−1^) is noticed in the FTIR spectra when the composite particles have a strong interaction. These characteristics strongly demonstrate the existence of functional carbonyl and carboxylic groups on the rGO flakes surface [33].

### 3.4. Dielectric Properties

**Figure 7:**
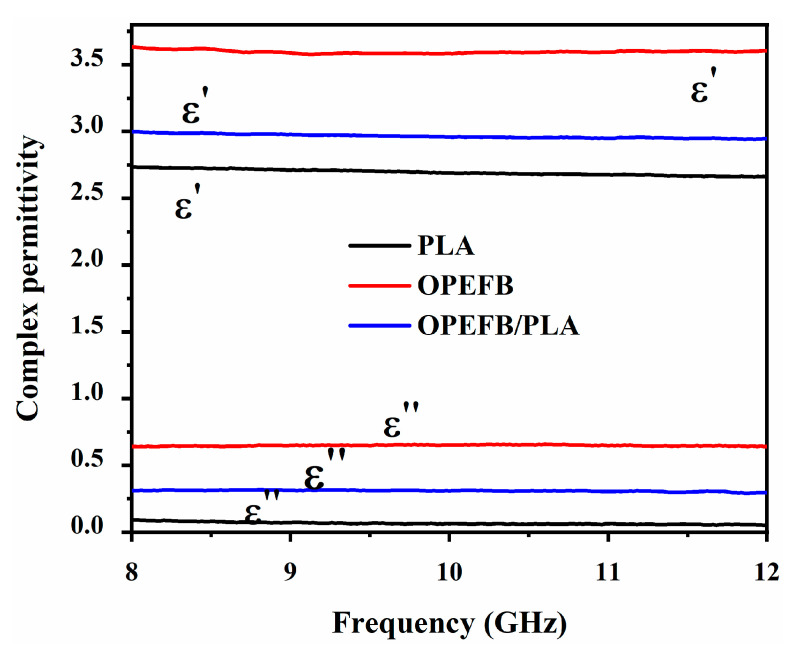
The variation of dielectric properties of OPEFB, PLA, and OPEFB/PLA composite with frequency.

The significant impacts that occur upon the interaction between a dielectric material and electric field are: surface transmittance, energy absorbance, and energy reflection. These impacts help to show the electrical properties associated with the relative permittivity. To analyze the dielectric properties of the OPEFB fiber, PLA, OPEFB/PLA matrix, and rGO/OPEFB/PLA composites compressed substrates were characterized for their real ($\varepsilon^{\prime }$) and imaginary ($\varepsilon^{\prime \prime }$) parts of the relative complex permittivity (*ԑ*^⁎^ = $\varepsilon^{\prime }$ − *j*$\varepsilon^{\prime \prime }$) at 8–12 GHz frequency range. Figure 7 shows the dielectric properties for the OPEFB fiber, PLA, OPEFB/PLA matrix. PLA has low complex permittivity due to the low polarization of the macromolecules. Adding fillers to the polymer will substantially boost the matrix’s low permittivity [18] since the polarization of filler/polymer interfaces (interfacial polarization), and filler can significantly contribute to the overall polarization of the composite. For all the samples, $\varepsilon^{\prime }$ and $\varepsilon^{\prime \prime }$ decreased as the frequency increased. The OPEFB fiber had the highest values of $\varepsilon^{\prime }$ and $\varepsilon^{\prime \prime }$ of 3.63 and 0.64 at 8 GHz, then slowly decreased to 3.48 and 0.59 at 12 GHz. The PLA had $\varepsilon^{\prime }$ and $\varepsilon^{\prime \prime }$ values of 2.74 and 0.09 at 8GHz, later reduced to 2.66 and 0.05 at 12 GHz. The OPEFB/PLA matrix also shows the same pattern where the values of $\varepsilon^{\prime }$ and $\varepsilon^{\prime \prime }$ at 8 GHz were found to be 3.01 and 0.31, then decreased to 2.94 and 0.96 at 12 GHz, respectively. A reduction in the orientation polarization at high frequencies resulted in a decrease in the $\varepsilon^{\prime }$ corresponding to the frequency [34].

**Figure 8:**
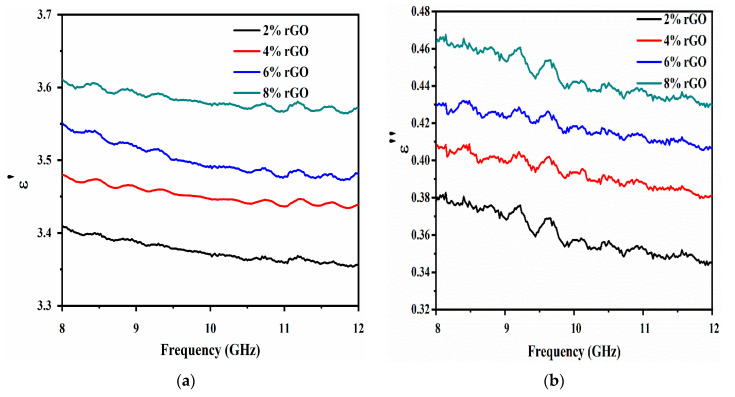
(**a**) Dielectric constant and (**b**) loss factor of rGO/OPEFB/PLA composites.

The effect of the 2–8% rGO filler loadings on the dielectric permittivity of rGO/OPEFB/PLA composites was studied, and the variation in $\varepsilon^{\prime }$ and $\varepsilon^{\prime \prime }$ values in the 8–12 GHz range are shown in Figure 8a,b. The real ($\varepsilon^{\prime }$) and imaginary ($\varepsilon^{\prime \prime }$) parts of the complex permittivity decreased as the frequency increased. The result showed that the microwave electric field affected the interaction of rGO/OPEFB/PLA composites with electromagnetic waves. Once the microwave frequency was raised, an electric field was generated that varied continuously. The various electrical field created polarization in the composites. Dipole moment slowly decreased in composites as frequency increased. Also, the dipole had a lesser duration to realign to the oscillating electric field [35]. $\varepsilon^{\prime }$ and $\varepsilon^{\prime \prime }$ values of the composites increased with higher loadings of rGO contents throughout the investigated frequency range. It can be seen that the least $\varepsilon^{\prime }$ (3.38) was measured for the 2% rGO filler, whereas the highest $\varepsilon^{\prime }$ (3.60) was measured for the 8% rGO filler. Also, the lowest $\varepsilon^{\prime \prime }$ (0.36) was obtained from the 2% rGO filler, whereas the highest $\varepsilon^{\prime \prime }$ (0.45) was measured for the 8% rGO filler. The increment of the dielectric properties can be attributed to interfacial polarization. The $\varepsilon^{\prime }$ of rGO/OPEFB/PLA composite is higher than the OPEFB/PLA matrix composite, which is attributed to widely available rGO polarization defects and interfacial polarization caused by OPEFB/PLA and rGO interface coupling [36]. The distribution of rGO particles was low at 2% rGO; therefore, their interaction with the OPEFB/PLA matrix is weak. When the concentration of rGO filler increases, the particles’ interaction within the matrix increased. The average polarization correlated with a cluster of particles is more robust than an individual particle due to improved composite inclusion dimensions and thus greater interfacial area [37], this results in higher average polarization, and hence, a higher contribution to the dielectric properties. However, it is observed that even at 8% rGO, the obtained permittivity values were lower than values obtained by Kashi et al. [38], where $\varepsilon^{\prime }$ and $\varepsilon^{\prime \prime }$ attained values of 11.17 and 0.87, respectively, for 6% GNP. A significant permittivity value difference attributed to the dispersion state of fillers in the matrix, which in turn depends on the affinity and interactions between the fillers and the polymer [39].

The $\varepsilon^{\prime \prime }$ part of the permittivity represents the energy dissipation. The grain boundaries had high resistivity at low frequencies and, due to the aggregation of electrons at the grain boundaries, a higher energy acquisition was needed for electron hopping, resulting in higher electrical energy loss. Conversely, the grain boundaries had low resistivity at high frequencies and thus less loss of electrical energy from electron hopping [40].

### 3.5. Shielding Effectiveness

**Figure 9:**
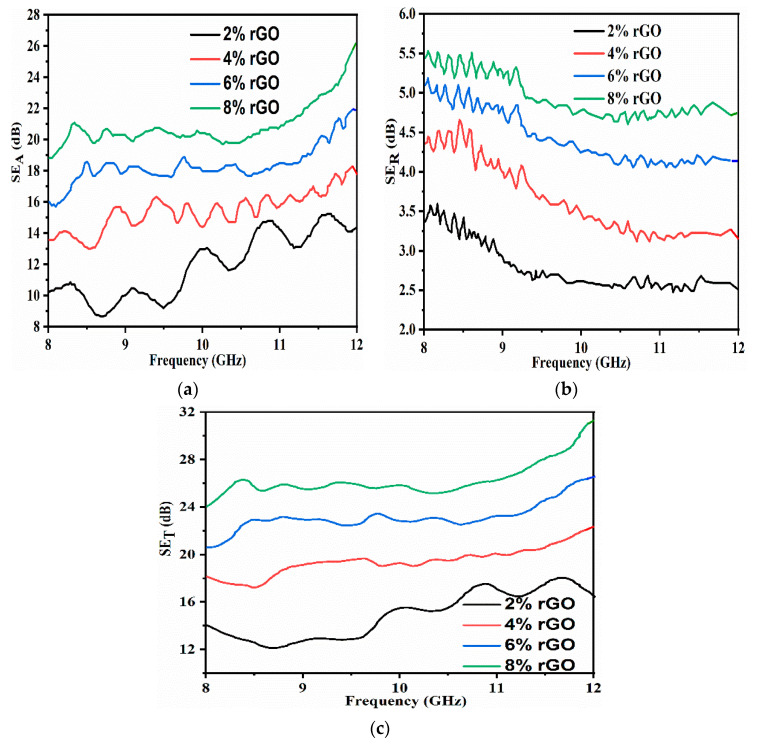
Variation of rGO/OPEFB/PLA composites (thickness of 6 mm) due to (**a**) absorption, (**b**) to reflection and, (**c**) total EMI shielding.

Figure 9a–c presents the frequency variation of rGO/OPEFB/PLA composite EMI shielding responses in the 8–12 GHz frequency range. Figure 9a reveals that the values of SE_A_ for the rGO/OPEFB/PLA composites increased significantly in frequency. The SE_A_ values of 6% rGO composites, for example, increased from 16.0 dB at 8 GHz to 20.8 dB at 12 GHz, whereas SE_R_ decreased from 5.2 dB at 8 GHz to 4.4 dB at 12 GHz as depicted in Figure 9b. It is also noteworthy that SE_A_ increases as rGO increased, and this result can be ascribed to the conductive mesh refinement. As the content of rGOs increases, their intercept with the polymer matrix increased, which leads to improvement of polarization and, therefore, absorbance. This can be attributed to the electron motion hysteresis under an alternating EM field, induced additional polarization relaxation processes that helped improve microwave absorption attenuation [41].

Figure 9c illustrates the SE_T_ of rGO/OPEFB/PLA composites. The results indicate that the addition of rGO enhanced the composites’ EMI shielding property, which increased with the rGO content increase. The increased polarity of all composites attributed to the rise in rGO filler concentration, which resulted in the enhancement of the shielding capacity by large numbers of mobile charging carriers, making the interaction with an external EM field easier. Moreover, the frequency dependence of SE_T_ is observed for a highly filled PLA matrix that exhibits a decreasing trend with increasing frequency. Similar behavior on other filled polymers was observed [42]. The summary of SE_T_ (dB) values of OPEFB/PLA matrix filled with 2%, 4%, 6%, and 8% rGO content at some selected frequencies such as 8, 10, 12 GHz is presented in Table 1.

## 4. Conclusions

In this study, chemically reduced graphene oxide (rGO) was synthesized from graphite powder using an ammonia solution method. The rGO was used as filler in PLA matrix reinforced with 100 µm OPEFB fiber to fabricate rGO/OPEFB/PLA composites, which enhanced complex permittivity values and excellent shielding effectiveness properties with increased filler content. The rGO/OPEFB/PLA composites were characterized for their dielectric properties at 8 to 12 GHz frequency range. The $\varepsilon^{\prime }$ and $\varepsilon^{\prime \prime }$ values of all the composites increased with increase in rGO content. The higher attenuation due to absorption and reflection (25.9dB and 5.49 dB) was obtained for the composite filled with 8 wt.% rGO content. A maximum SE_T_ value of 31.2 dB was achieved for the composite with 8% rGO filler. The markedly enhanced SE_T_ obtained with the addition of OPEFB fiber is a promising new and secure method for the application of rGO-filled composites to microwave absorbing applications. The study reveals that rGO/OPEFB/PLA composites are potential candidates for EMI shielding applications in the area where lightweight material is required.

## Figures and Tables

**Table 1:** Variation in SE_T_ (dB) at specified frequencies.

Frequency (GHz)	2%	4%	6%	8%
8	12.7	17.9	22.5	25.4
10	15.6	19.6	22.9	26.1
12	16.1	21.3	26.4	31.2
